# The Grand Convergence: Closing the Divide between Public Health Funding and Global Health Needs

**DOI:** 10.1371/journal.pbio.1002363

**Published:** 2016-03-02

**Authors:** Mary Moran

**Affiliations:** Executive Director, Policy Cures, Sydney, Australia

## Abstract

The Global Health 2035 report notes that the “grand convergence”—closure of the infectious, maternal, and child mortality gap between rich and poor countries—is dependent on research and development (R&D) of new drugs, vaccines, diagnostics, and other health tools. However, this convergence (and the R&D underpinning it) will first require an even more fundamental convergence of the different worlds of public health and innovation, where a largely historical gap between global health experts and innovation experts is hindering achievement of the grand convergence in health.

## Abstract 2

Are public health organizations unwittingly delaying health gains? This Perspective assesses the gap between public health experts and those who can deliver the tools they need.

The Global Health 2035 report notes that the “grand convergence”—closure of the infectious, maternal, and child mortality gap between rich and poor countries—is dependent on research and development (R&D) of new drugs, vaccines, diagnostics, and other health tools. New tools alone are estimated to deliver a 2% decline each year in the under-5 mortality rate, maternal mortality ratio, and deaths from HIV/AIDS and tuberculosis (TB) [1].

However, this convergence (and the R&D underpinning it) is unlikely unless we first have an even more fundamental convergence of the parallel worlds of public health and innovation. At the moment, these worlds are often disconnected, with major gaps to be bridged at both the intellectual and practical levels before we can truly reach a grand convergence in health.

## Promising Language

The new Sustainable Development Goals (SDGs), which will replace the Millennium Development Goals (MDGs) at the end of 2015, set out ambitious aims including—by 2030—“ending the epidemics of AIDS, TB, malaria and NTDs,” “ending preventable deaths of newborns and under-5 children,” and “reducing maternal mortality to less than 70 per 100,000 live births” [2].

Global health agencies explicitly acknowledge that these goals and strategies will need R&D of new tools and have reflected this in global plans for AIDS, TB, malaria, and other diseases. The Global Plan to Stop TB (2011–2015) states that “without sufficient investment in the development of new diagnostic methods, anti-TB drugs, and vaccines, we will not achieve the Partnership’s goal of eliminating the disease as a public health problem by 2050”[3]. The World Health Organization (WHO) notes that “it is unlikely that the ambitious HIV targets set for 2020 and 2030 can be achieved if we rely only on existing HIV technologies” [4].

A list of the 145 “missing” drugs, vaccines, diagnostics, microbicides, vector control agents, and technologies needed to reach these goals has also been compiled by global health experts: this list forms the basis of the G-FINDER survey of R&D investments in global health [5,6]. Box 1 provides further information about G-FINDER.

**Box 1. G-FINDER**

G-FINDER is an annual survey of investment into product R&D for neglected diseases of the developing world. It covers 34 diseases and includes data from over 200 public, private, and philanthropic funders.

In 2013,

- US$3.2 billion was invested into R&D for neglected diseases
- 70% of R&D funding was for HIV/AIDS, TB, and malaria
- US$88 million was invested into R&D of reproductive health products for the developing world (surveyed for the first time in 2013)
- 90% of global R&D funding was from science agencies, the private sector, and philanthropies

## The Gap between Words and Deeds

Yet, in a somewhat Kafkaesque disconnect, these statements by the global health community of their support for—and belief in—the need for R&D of new global health tools are not reflected by their actions in the real world. The examples of malaria and postpartum haemorrhage are symptomatic.

Malaria mortality rates have fallen globally by 47% since 2000 (and in African children by 58% [7]) because of the potent combination of expanded malaria programmes and new tools. Programmes have replaced chloroquine with artemisinin combination therapies; microscopy with rapid diagnostic tests to a large degree; and short-acting insecticide-treated nets (ITNs), which needed retreatment after three washes, with re-engineered long-acting ITNs that last 20 washes or three years. The malaria community has now signed up for a global malaria elimination and eradication agenda that will require further new tools, including diagnostics for low-level parasitaemia and new vector control agents to counter emerging resistance. Yet, only two international development agencies invested more than US$5 million into malaria R&D in 2013 (United States Agency for International Development [USAID] with US$6 million, and United Kingdom Department for International Development [DFID] at US$28 million).

Postpartum haemorrhage (PPH) is responsible for 30% of maternal mortality in Asia and sub-Saharan Africa (over 3 million disability-adjusted life years [DALYs] each year), largely because the treatment (oxytocin) requires refrigeration and skilled health worker administration—unrealistic demands for many births in poor countries. In 2008, it was reported that only one new class of drug had been licensed for obstetric conditions in the previous 20 years [8]. Meeting the SDG target of a 70% reduction in maternal mortality should, logically, include new tools for PPH, yet development agencies provided only US$1.1 million in total to R&D for PPH in 2013 [9].

### A Funding Gap

Development agencies do spend on research (for example, the UK, Swedish, Swiss, Canadian, and Australian agencies spend between 2%–3% of their programmatic aid budgets in research and between 10%–40% of this on health research [10]), but their focus is often on operational and field research, not technology innovation or product development.

In 2013, only 11 of the world’s development agencies funded any global health R&D, with eight of these investing less than US$10 million per year. Three agencies—USAID, DFID, and the Directorate-General for International Cooperation (DGIS)—accounted for 86% (US$190 million) of the total (Table 1).

**Table 1:** Development agency funding for global health R&D (2013).

Agency	US$
United States Agency for International Development (USAID)	90,368,787
UK Department for International Development (DFID)	73,848,372
Dutch Ministry of Foreign Affairs–Directorate General for International Cooperation (DGIS)	27,649,666
Irish Aid	9,295,586
Royal Norwegian Ministry of Foreign Affairs and/or Norwegian Agency for Development Cooperation (NORAD)	5,108,050
Swiss Agency for Development and Cooperation (SDC)	5,092,013
Canadian Department of Foreign Affairs, Trade, and Development	5,047,848
Danish Ministry of Foreign Affairs and/or Danish International Development Agency (DANIDA)	4,503,645
French Development Agency, Agence Française de Développment (AFD)	1,593,529
Swedish International Development Cooperation Agency (SIDA)	726,590
Flemish International Cooperation Agency (FICA)	296,904
Total	223,530,990

Source: Policy Cures. G-FINDER public search tool. 2015. Available from https://gfinder.policycures.org/PublicSearchTool/

In reality, the vast bulk of public funding for the new tools needed by public health programmes comes not from the public health field but from domestic science and research agencies (83% of public funding), with aid agencies providing only 10%. The remainder comes from multilaterals and other government funders. In 2013, 48 science and technology agencies provided US$1.7 billion for global health R&D (eight times more than development agencies), with 70% of this coming from the US National Institutes of Health (NIH) and 14 other science agencies providing US$10 million or more to global health research (Table 2) [9].

**Table 2:** Top 15 science agencies funding global health R&D (2013).

Agencies	US$
US National Institutes of Health (NIH)	1,247,608,824
European Commission	122,584,302
Inserm–Institute of Infectious Diseases	61,464,777
UK Medical Research Council (MRC)	47,578,600
Indian Council of Medical Research (ICMR)	39,864,197
Australian National Health and Medical Research Council (NHMRC)	27,571,547
French National Agency for Research on AIDS and Viral Hepatitis (ANRS)	20,754,656
German Research Foundation (DFG)	18,321,626
German Federal Ministry of Education and Research (BMBF)	17,152,276
Brazilian Research Support Foundation of the State of Amazonas (FAPEAM)	15,128,596
Canadian Institutes of Health Research (CIHR)	13,282,940
South African Department of Science and Technology (DST)	10,989,863
Argentinean Ministry of Science, Technology, and Productive Innovation	10,835,866
Swiss National Science Foundation (SNSF)	10,595,557
Indian Department of Biotechnology, Ministry of Science, and Technology (DBT)	9,939,191

Source: Policy Cures. G-FINDER public search tool. 2015. Available from https://gfinder.policycures.org/PublicSearchTool/

Some might argue that this doesn’t matter—if science agencies provide the R&D funding, then well and good. However, science funding differs from development agency funding in ways that crucially matter for global health. Science funding is shaped by biomedical research paradigms rather than global health paradigms; it has a predominantly domestic rather than an international focus; and it is often investigator driven, rather than being linked to development priorities and strategies—for instance, while new tools for post-partum haemorrhage (PPH) are a development priority, they receive very little science funding (science agencies reported PPH grants of only US$81,476 in 2013) [6]. Science funding is also skewed towards basic research rather than product development. In 2013, aid agencies invested 88% of their funding into product development and less than 1% into basic research; by comparison, science agencies invested 35% into basic research—and close to 40% during the global financial crisis when spending at home was a priority.

### A Strategy Gap

The innovation disconnect is not restricted to funding; it also appears at the most fundamental level of global health planning. The SDGs include 17 goals and 169 targets for development in the next 15 years: these include one health goal, which has nine health-related targets. Most of these nine health targets cannot be achieved with today’s tools, including for HIV, TB, and reproductive health as noted above, yet neither the health goal nor any of its nine targets mention R&D. Indeed, health R&D is mentioned only once in the SDG documents, in one of the four “means of implementation” targets appended to the health goal, and then it omits key areas of need (diagnostics, microbicides, reproductive health tools, vector control agents, and platform technologies are all missing) and is conflated with language on access to existing medicines. Global health agencies, including WHO, the United Nations Population Fund (UNFPA), the UN Children's Fund (UNICEF), the UN Office on Drugs and Crime (UNODC), the Joint UN Programme on HIV and AIDS (UNAIDS), UN Women, the UN Environment Programme (UNEP), and others, were asked to recommend health-specific indicators for the SDG process, but R&D was not included (or mentioned) in either their long list of 40 indicators or in their subsequent shortlists [11,12].

**Fig 1:**
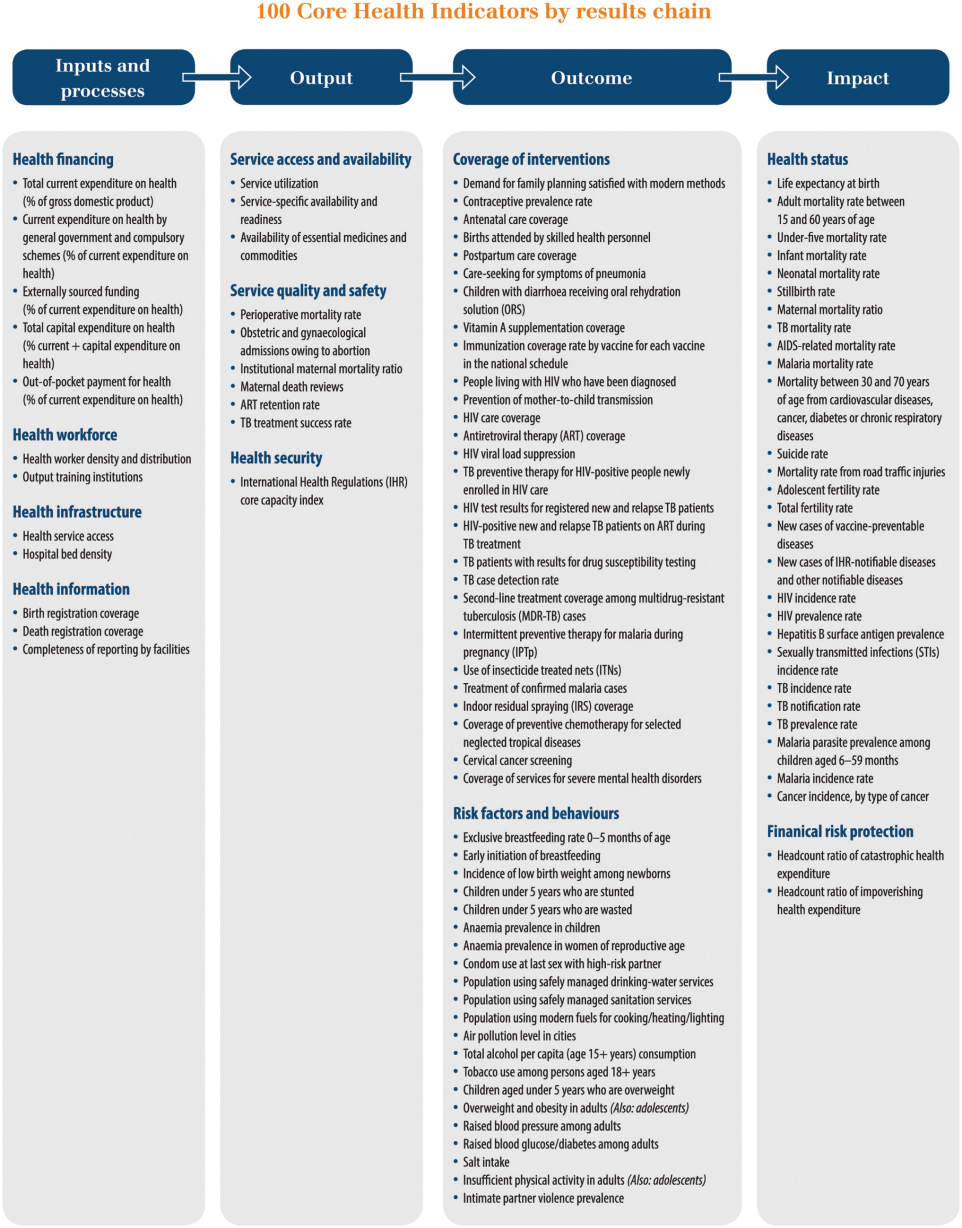
Global Reference List of 100 Core Health Indicators (by results chain). Source: Reprinted from Global Reference List of 100 Core Health Indicators 2015, World Health Organization, 100 Core Health Indicators 2015, p.20, Copyright 2015 [cited 2015 August 13]. Available from http://www.who.int/healthinfo/indicators/2015/en/.

In November 2014, 19 global health agencies, led by the director-general of WHO, compiled a Global Reference List of 100 Core Health Indicators “that the global community prioritizes for the purposes of monitoring global progress, maintaining programme support, and advocating for resources and funding” (Fig 1) [13]. R&D was again absent, including from the inputs needed to achieve the desired health impacts; instead, these inputs focused on traditional public health issues such as workforce, infrastructure, health data, and financing for health services.

Unless there is a dramatic change, and a far greater focus on new tools to manage common causes of mortality such as PPH (27% of maternal deaths) [14] and neonatal infections and asphyxia (59% of neonatal deaths) [15], the SDG maternal and child health goals are even less likely to be met than their MDG counterparts. This is of particular concern given that the maternal health goal (Goal 5) is still the furthest from being reached of any of the MDGs [16].

## Why the Divide?

Stakeholders on both sides of the innovation–public health divide are experienced, dedicated people whose focus is improving health in the developing world. So, why this gap? The reasons seem largely historical, making it hopeful that we are now ready for change.

### Innovation Starvation

**Fig 2:**
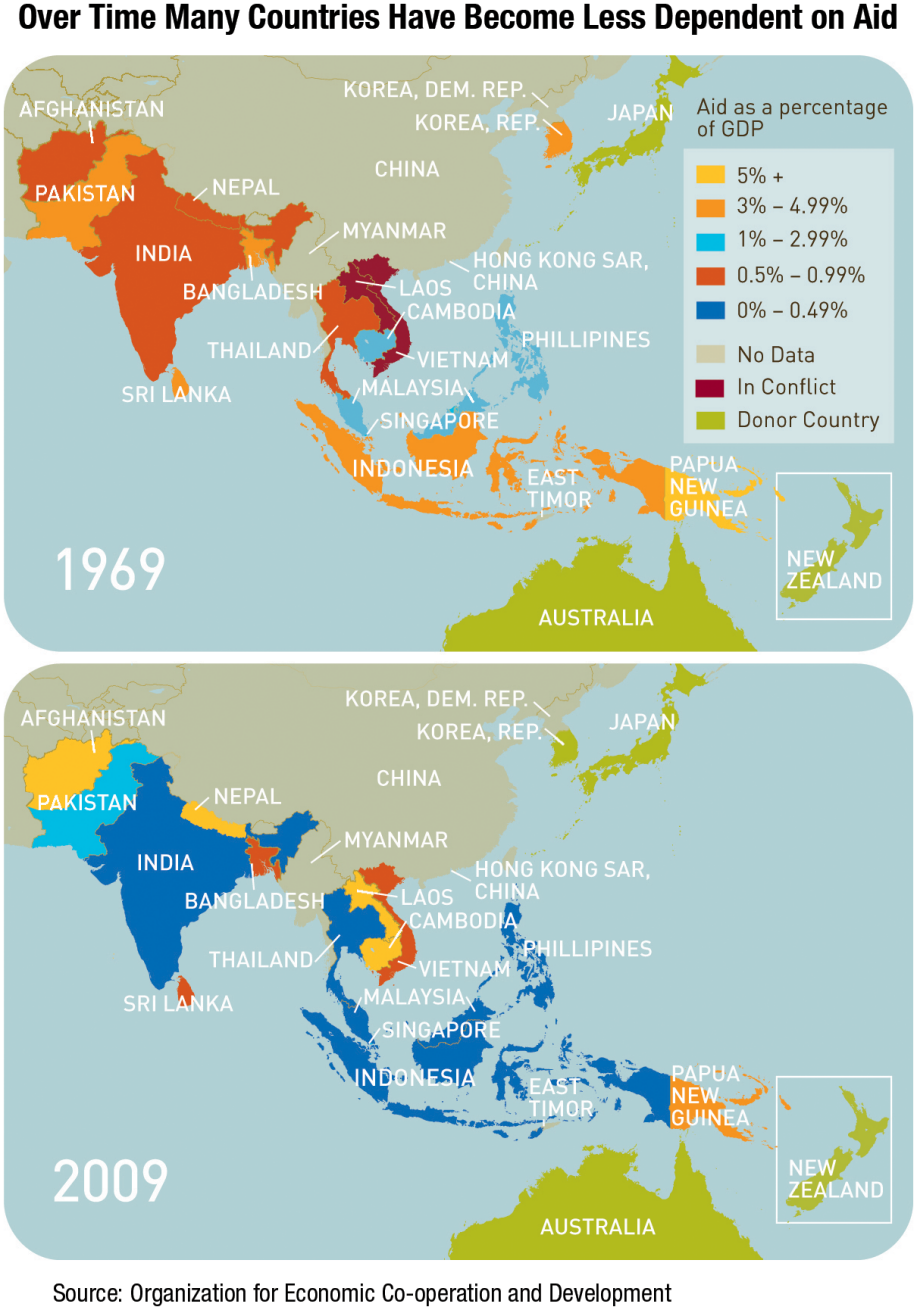
Decrease in aid dependency. Source: Gates, B. Innovation with impact: financing 21st century development. Cannes, France; 2011 [cited 2015 August 13]. Available from http://www.gatesfoundation.org/What-We-Do/Global-Policy/G20-Report.

For many decades, innovation was not a subject for public health. Aid dollars had to stretch across many more countries and to support many more people living on under US$1.25 a day (Fig 2) [17]. The imperative for public health programmes was to “make do with what we have,” deploying available low-cost tools for the greatest good: historically, around 95% of drugs on the WHO’s Essential Medicines List were older off-patent medicines [18]. New, more expensive innovations were seen as something for Western health systems, with long delays in their developing country introduction accepted as the norm. The first Hib vaccine was registered in 1985 but not recommended by WHO for developing country use until 2006, and WHO officials explicitly advocated “restraint in the adaptation of new technologies by nonindustrialised nations” [19].

In many cases, there were no new technologies to hanker for: only 16 new chemical entities were marketed for tropical diseases and TB between 1975 and 1999 [20]. Even today, TB relies on first-line drugs developed between 1952 and 1963; and first-stage sleeping sickness is still treated with five weeks of suramin (1920) or daily injections of pentamidine (1940). As a result, the public health community became inured to insufficient resources and old tools, and—beyond the medical research fraternities—often had limited exposure to product innovation and innovators.

### A False Dichotomy

These historical factors have sometimes led to an unproductive tension between global health and innovation, with an implicit (and even explicit) view that scarce health funds should be spent on programmes and service delivery, not on developing new tools. In 2014, advocates from the European Union (EU), the US, and Australia identified that domestic global health academics, concerned about competition for funding, were one of the key barriers to securing new resources for product development [21]—arguing, for instance, that the reduction in malaria deaths since 2000 supported the case for investment in programme delivery (rolling out bed nets), not for product development [22]. These statements repeat the WHO argument, from as far back as 1994, that “there is a danger that their (new technologies) introduction would divert attention and money away from the real issues” [23].

This “us or them” dichotomy is unhelpful and also unfounded: a health programme is only as good as the tools it uses, and the tools are only as useful as the programme that delivers them. Innovation doesn’t compete with programme funding; rather, it frees up funding by cutting disease burdens and programmatic costs. Vaccine vial monitoring devices are estimated to have saved immunisation programmes and procurement agencies more than US$140 million in wasted vaccines in the past decade [24]. Health systems have seen huge savings as measles, diphtheria, whooping cough—and more recently polio, *Haemophilus* flu, and meningitis A—have diminished. These are all now vaccine preventable, while better malaria tools in expanded malaria programmes have averted an estimated 274 million malaria cases between 2001 and 2010. The cost of R&D, which seems so high during the innovation process, becomes vanishingly small over decades of use and millions of patients and even more so when set against the saving of lives and health dollars that is the hallmark of good innovation.

### Picking up the Baton

A final issue, and one that extends beyond the global health community to governments as a whole, is that we have entered a new world when it comes to R&D for neglected areas. Traditionally, when new medicines are needed, governments provide science funding for early academic research, and industry then commercialises these leads as new products for patients. However, there is no commercial industry for neglected diseases (hence their title), and there has not been since infections like malaria and TB receded from the West after WWII.

When it comes to the neglected and reproductive health needs of the developing world, governments, including both science and development agencies, must therefore drive the entire R&D process, from basic research through to and including final product development. Science agencies have more than kept up their end of the deal, providing over 80% of public funding for global health R&D and in particular for basic research, as noted earlier, but product development has been badly underfunded, with development agencies in particular failing to pick up the baton. Philanthropy has been generous in making up some of the shortfall, providing over half of all funding to the Product Development Partnerships [5] that have created over 70% of all new drugs and vaccines registered between 2000 and 2011 [25], but philanthropists cannot bear the entire funding burden, nor should their priorities be the dominant force in determining which global health products are developed.

A further area in which governments could make a difference is in improving and aligning regulatory requirements or reviews for new global health products to avoid expending valuable funds (and valuable patient time) on often repetitive, sequential approvals in each country.

In 2012, a WHO official, when asked why malaria R&D was not included in the draft Ministerial Communique of the Asia-Pacific Malaria Summit, responded “because companies look after that” [26]. Unless this belief changes at the most fundamental levels of the global health community, patients may wait even more decades for the better and more effective medicines they deserve.

## Solutions

If we are to see a grand convergence, global health and development agencies will need to address the structural R&D gap that exists between discovery and delivery today.

We recommend that global health and development agencies do the following:

1. Energetically advocate for a health R&D indicator to be included in the final list of (likely around) 120 SDG indicators. Without R&D and a metric to drive it, the health goal cannot be achieved, as noted by health agencies themselves.
2. Define their R&D needs (as is happening in this series of papers), rather than waiting to see what others have developed.
3. Urgently start providing serious global health R&D funding not just for traditional operational and health systems research but to global health R&D and in particular to new product development.
4. Rapidly increase their innovation expertise in order to make effective R&D prioritisation and funding decisions. In the short- to mid-term, this means bringing in or partnering with innovation and product development expertise, including from industry. Conflict of interest will be an issue, but there are better ways to manage this than by excluding innovation expertise from the room. Good innovation policy and well-targeted investments require public health expertise but cannot be built on this alone.
5. Engage far more closely with science agencies, to secure better sequencing of their activities and a better strategic alignment between what the science community does and what the global health community needs. To this end, the science community could also helpfully communicate more in the language of public health and less in the language of antibodies and targets.

With greater familiarity and knowledge, innovation can become an integral part of the global public health paradigm; built on the recognition that, rather than competing for aid dollars, innovation decreases the demands on aid funding by shrinking diseases ranging from measles, diphtheria, and polio to river blindness, meningitis, and malaria. As global health experts begin to routinely include not only today’s knowledge but tomorrow’s tools in their vision and actions, we can indeed hope for a grand convergence.

## Abbreviations

AFD: Agence Française de Développment
DALY: disability-adjusted life year
DANIDA: Danish International Development Agency
DFID: Department for International Development
DGIS: Directorate General for International Cooperation
EU: European Union
FICA: Flemish International Cooperation Agency
ITN: insecticide-treated net
MDG: Millennium Development Goal
NIH: United States National Institutes of Health
NORAD: Norwegian Agency for Development Cooperation
PPH: postpartum haemorrhage
R&D: research and development
SDC: Swiss Agency for Development and Cooperation
SDG: Sustainable Development Goal
SIDA: Swedish International Development Cooperation Agency
TB: tuberculosis
UNAIDS: Joint United Nations Programme on HIV and AIDS
UNEP: United Nations Environment Programme
UNFPA: United Nations Population Fund
UNICEF: United Nations Children's Fund
UNODC: United Nations Office on Drugs and Crime
USAID: United States Agency for International Development
WHO: World Health Organization
