# Correction: The Grand Convergence: Closing the Divide between Public Health Funding and Global Health Needs

**DOI:** 10.1371/journal.pbio.1002439

**Published:** 2016-04-01

**Authors:** The PLOS Biology Staff

## Notice of Republication

This article was republished on March 15, 2016, to correct errors in the article layout that were introduced during the typesetting process. Specifically, the abstract should have only contained a single paragraph, however the first two paragraphs of the main article text were also included in this. The publisher apologizes for the errors. Please download this article again to view the correct version. The originally published, uncorrected article and the republished, corrected articles are provided here for reference.

## Supporting Information

S1 File Originally published, uncorrected article. (PDF)

S2 File Republished, corrected article. (PDF)
